# The recurrent campylobacteriosis epidemic over Christmas and New Year in European countries, 2006–2014

**DOI:** 10.1186/s13104-017-2587-8

**Published:** 2017-07-11

**Authors:** Philipp Justus Bless, Claudia Schmutz, Daniel Mäusezahl

**Affiliations:** 1 Swiss Tropical and Public Health Institute, Socinstrasse 57, P. O. Box 4002, Basel, Switzerland; 2 University of Basel, Petersplatz 1, P. O. Box 4001, Basel, Switzerland

**Keywords:** *Campylobacter*, Infectious disease surveillance, Europe, Seasonality, The European Surveillance System (TESSy)

## Abstract

**Objective:**

Campylobacteriosis is the most frequently reported foodborne disease in Europe with a notification rate of 71 per 100,000 population in the European Union in 2014. Surveillance data show a clear seasonality whereby case numbers peak during summer months in entire Europe and at the turn of the year, especially in Germany and Switzerland. A detailed description of European surveillance data by country at the turn of the year was missing so far. The objectives of the presented work were to describe national surveillance data of The European Surveillance System for 14 countries during winter times and to generate hypotheses for the observed seasonality of campylobacteriosis cases.

**Results:**

The analysis included 317,986 cases notified between calendar weeks 45 and 8 of winter seasons 2006/2007–2013/2014. Winter peaks in weekly case notifications and notification rates were observed for Austria, Belgium, Finland, Germany, Luxembourg, The Netherlands, Switzerland and Sweden while for Denmark, France, Ireland, Italy, Norway and the United Kingdom no unusual increase was observed. Generally, weekly notification rates peaked in calendar week 1 or 2 after a strong decline in the last week of December and reached values of a multiple of the observed notification rates in the weeks before or after the peak e.g. up to 6.5 notifications per 100,000 population per week in Luxembourg. Disease onset of cases notified during winter peaks occurred predominantly in calendar weeks 52 and 1 and point towards risk exposures around Christmas and New Year. The consumption of meat fondue or table top grilling poses such a risk and is popular in many countries with an observed winter peak. Additionally, increased travel activities over the festive season could foster campylobacteriosis transmission. Surveillance artefacts (e.g. reporting delays due to public holidays) should be excluded as causes for country-specific winter peaks before investigating risk exposures.

**Electronic supplementary material:**

The online version of this article (doi:10.1186/s13104-017-2587-8) contains supplementary material, which is available to authorized users.

## Introduction

Since 2005, human campylobacteriosis has been the most frequently reported foodborne bacterial gastrointestinal disease in Europe. Case numbers are increasing [1]. In 2014, around 237,000 cases were reported by 26 European Union (EU) member states corresponding to a notification rate of 71 per 100,000 population [1]. European campylobacteriosis surveillance data show a clear seasonal trend [2]. The number of notified cases starts to increase drastically in April and peaks during summer, between June and August [2]. The lowest numbers of cases are notified in February and March [2]. In the campylobacteriosis surveillance data of the EU, in particular of Germany, and of Switzerland, an additional seasonal peak between late December and early January, the so-called winter peak, has been described [1, 3, 4]. The monthly incidence in Germany peaks in January [3] and case numbers in Switzerland increase during the last week of December and the 1st week of January [4].

Our investigation of the winter peak in Switzerland identified the consumption of meat fondue as main risk factor, especially if served with chicken [5]. Meat fondue is traditionally consumed on Christmas day and on New Year’s Eve in Switzerland and is also a popular dish at New Year’s Eve in Germany and Luxembourg [5, 6]. A detailed description of European *Campylobacter* surveillance data at the turn of the year is missing so far and hence, it is unknown in which other European countries winter peaks in notification data occur. This study analyses European country-specific surveillance data at the turn of the year from 2006 to 2014, to determine if winter peaks as observed in Switzerland and Germany also occur in other European countries and to generate hypotheses for the seasonal patterns.

## Main text

### Analysis of *Campylobacter* surveillance data

This study considered Switzerland, Germany and neighbouring countries (Austria, Belgium, France, Italy, Luxembourg, The Netherlands), countries of the British Isles (Ireland, United Kingdom) and Nordic countries (Denmark, Finland, Norway, Sweden). For EU member states, case-based notification data on laboratory-confirmed *Campylobacter* infections from 2006 to 2014 originated from The European Surveillance System (TESSy)—an indicator-based surveillance database for communicable diseases hosted by the European Centre for Disease Prevention and Control (ECDC) [7]. Surveillance data from the National Notification System for Infectious Diseases on laboratory-confirmed campylobacteriosis cases notified between 2006 and 2014 were used for Switzerland. Our previous analysis of Swiss notification data on *Campylobacter* showed that the winter peak is rather a short-term phenomenon and better observable in weekly than monthly notification data [4]. Therefore, we performed a descriptive analysis of country-specific weekly notification data focusing on the period of calendar weeks 45 to 8.

A total of 1,530,564 campylobacteriosis case notifications were received from TESSy. For 147 case notifications or 0.03% of all United Kingdom notifications no information on the week of notification was available. Hence, they were excluded from further analyses. In 2006 and 2007 German notification data were reported on a monthly basis leading to the exclusion of 118,142 case notifications. We additionally excluded 848 case notifications with a notification date in 2006 or 2014 but belonging to calendar week 52 of 2005 or calendar week 1 of 2015. For Italy no notification data from 2006 until mid-2008 were available. A total of 317,986 cases notified between calendar weeks 45 and 8 of the winter seasons 2006/2007–2013/2014 were analysed including 16,237 campylobacteriosis cases from Switzerland.

Weekly notification rates were calculated using annual country-specific population numbers as per 1st of January for each corresponding winter season from the Eurostat database [8]. The Dutch and French sentinel surveillance systems do not cover the whole population. We used the estimated population coverage for *Campylobacter* surveillance of 52% (The Netherlands) and 20% (France) [2] to calculate population numbers for the calculation of weekly notification rates. The population coverage of *Campylobacter* sentinel surveillance in Belgium and Italy is unknown and, hence, only case numbers were used. The sum of case numbers and the median of notification rates over all winter seasons are presented for each calendar week by country. Additionally, dates of disease onset or diagnosis were analysed to assess possible reporting delays. In the Additional file 1 case numbers and notification rates per calendar week for each winter season and country are presented.

### Seasonal patterns of campylobacteriosis

**Fig. 1:**
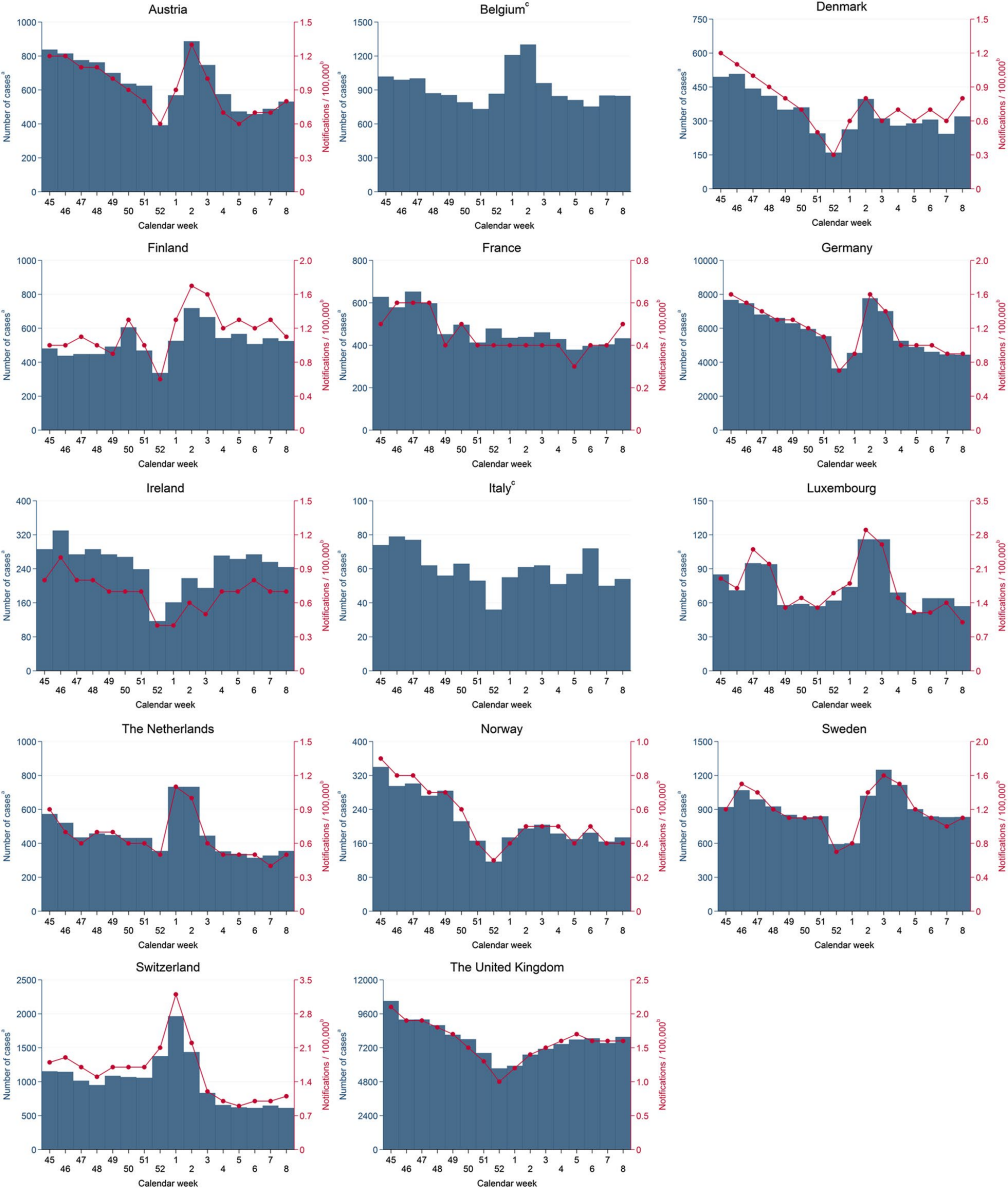
Number of case notifications and weekly notification rates per 100,000 population for campylobacteriosis in selected European countries, winter seasons 2006/2007–2013/2014. ^a^Sum of weekly notifications from winter seasons 2006/2007–2013/2014 (Germany and Italy: 2008/2009–2013/2014). ^b^Weekly notifications per 100,000 population = median of weekly notification rates from winter seasons 2006/2007–2013/2014 (Germany 2008/2009–2013/2014). ^c^Sum of weekly notifications only as coverage of surveillance system unknown. Note: Scales of y-axes differ between countries

The sum of case notifications and the median of notification rates by calendar week over all years increased at the end of December or beginning of January for Austria, Belgium (case notifications only), Finland, Germany, Luxembourg, The Netherlands, Sweden and Switzerland and decreased towards the end of January (Fig. 1). Winter peaks in terms of median notification rates were most pronounced in Luxembourg and Switzerland with peak rates of 2.9 and at 3.2 per 100,000 population, respectively (Table 1). Less pronounced winter peaks were observed in The Netherlands and Austria with peak rates of 1.1 and 1.3 per 100,000 population, respectively. The sum of weekly case notifications in Belgium peaked in week 2. For the other countries (Denmark, France, Ireland, Italy, Norway and the United Kingdom) no unusual increase during the winter season was observed (Fig. 1). A common characteristic of most countries was that the sum of case numbers and median notification rates were lowest at the end of December in week 52.

**Table 1:** Winter peaks of campylobacteriosis case notifications as median notification rate and sum of case notifications over all winter seasons, 2006/2007–2013/2014

Country	Calendar week of peak	Median notification rate^a^	Sum of case notifications
Austria	2	1.3	887
Belgium	2	N/A	1302
Finland	2	1.7	719
Germany	2	1.5	8807
Luxembourg	2	2.9	116
The Netherlands	1	1.1	733
Sweden	3	1.6	1250
Switzerland	1	3.2	1964

*N/A* not applicable

^a^Rate per 100,000 population

The weekly case numbers and notification rates of winter peaks varied in each country by year (Additional file 1). The most distinct winter peak with a weekly notification rate of 6.5 per 100,000 population was observed in Luxembourg during the winter season 2013/2014 (Table 2). Peak rates in other countries ranged from 1.8 in Germany and The Netherlands to 4.5 notifications per 100,000 population in Switzerland. From the beginning of the observation period in 2006/2007–2013/2014 peak case numbers and notification rates increased for Belgium, Germany, Luxembourg, The Netherlands, Sweden and Switzerland (Table 2). A more than threefold increase was observed for Luxembourg and a twofold increase for Switzerland. For Austria and The Netherlands winter peaks in 2006/2007 had higher peak rates compared to the subsequent years but afterwards peak rates started to increase discontinuously. In Austria peak rates increased by 45% from 1.1 to 1.6 notifications per 100,000 population between 2011 and 2014. The highest rate of the winter peak 2007/2008 in The Netherlands was 0.7 per 100,000 population and increased to twice this rate in 2013/2014.

**Table 2:** Weekly peak notification rates over winter seasons and changes of weekly peak notification rates between 2006/2007 and 2013/2014 winter seasons

Country	Maximum weekly notification rate of all winter peaks			Maximum weekly notification rate of winter peak 2006/2007		Maximum weekly notification rate of winter peak 2013/2014		Change of maximum weekly notification rates (2006/2007–2013/2014) (%)
	Notification rate^a^	Calendar week	Year	Notification rate	Calendar week	Notification rate	Calendar week	
Austria	1.9	2	2007	1.9	2	1.6	3	−15.8
Finland	3.1	50	2007	1.4	3	1.7	3	+21.4
Germany	1.8	2	2014	1.7^b^	3^b^	1.8	2	+5.9
Luxembourg	6.5	3	2014	1.9	2	6.5	3	+242.1
The Netherlands	1.8	1	2012	1.2	2	1.4	2	+16.7
Sweden	2.0	3	2008	1.5	3	1.5	3	0.0
Switzerland	4.5	1	2012	1.7	2	3.6	1	+111.8

^a^Rate per 100,000 population

^b^Winter peak 2008/2009

The Nordic countries Denmark and Norway exhibited no specific dynamics in the annual notification data on a regular basis (Additional file 1). However, Danish weekly case numbers and notification rates showed irregular increases resembling a winter peak during some winter seasons. In Norway case numbers and notification rates generally decreased around calendar weeks 51 and 52 and were sometimes slightly increased in calendar weeks 1, 2, or 3.

**Fig. 2:**
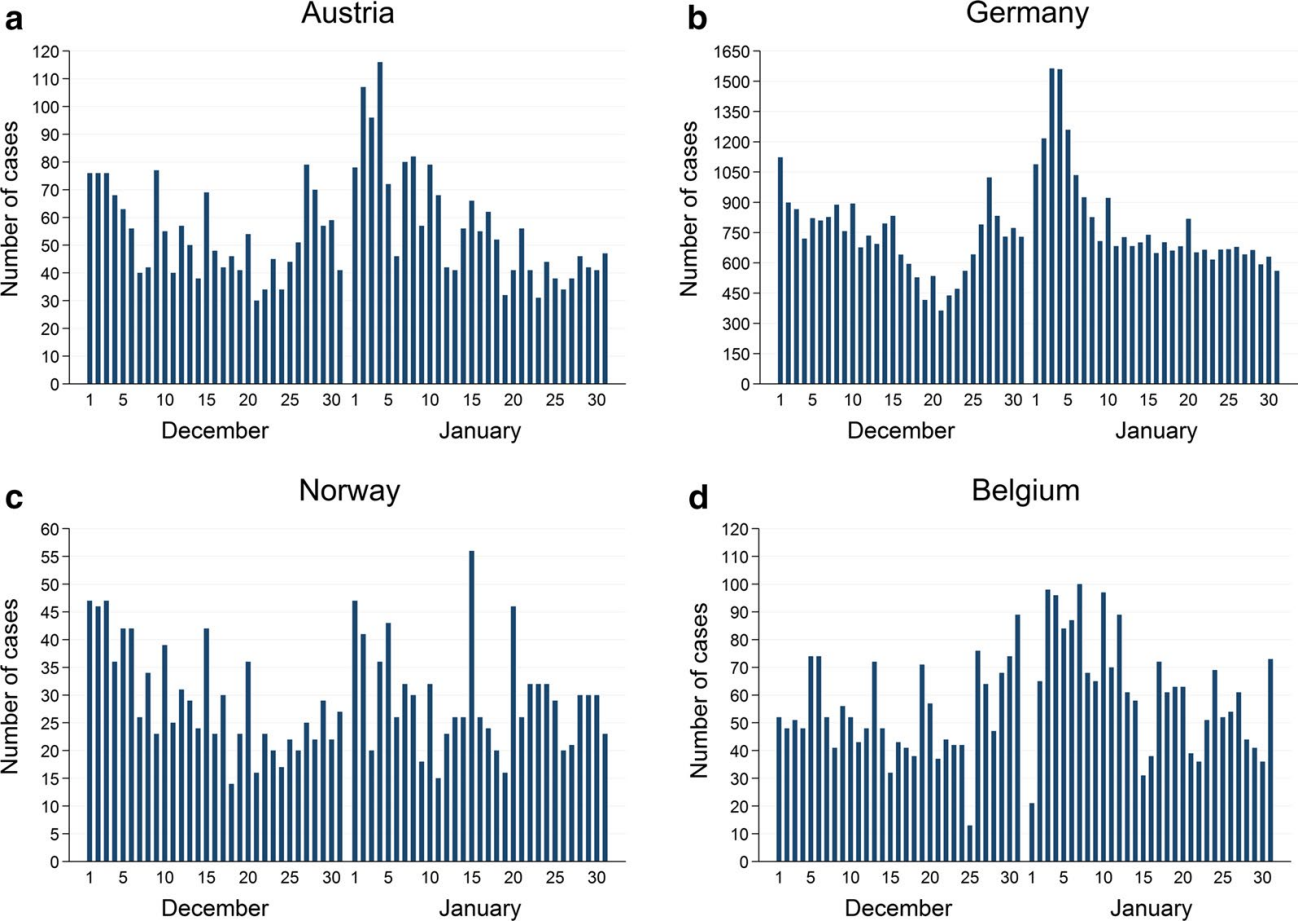
Sum of case notifications between 1st December and 31st January. **a** Austria by daily disease onset, winter seasons 2008/2009–2013/2014. **b** Germany by daily disease onset, winter seasons 2008/2009–2013/2014. **c** Norway by daily disease onset, winter seasons 2006/2007–2013/2014. **d** Belgium by daily diagnoses, winter seasons 2011/2012–2012/2013

Possible reporting delays were assessed for countries with observable winter peaks and for which dates of disease onset or dates of diagnosis were available (Austria, Belgium, Germany and Norway). Numbers of disease onset or diagnosis were summed up over all years per day and are depicted in Fig. 2. In Austria, Germany and Norway the daily numbers of disease onset peaked in the first week of January and to a smaller extent already in the last week of December. Peaks of disease onset dates occurred a few days to 1 week before winter peaks observed in actual notification data. The number of diagnoses in Belgium started to increase at the end of December and decreased after the 2nd week of January.

Our analysis of notification data shows that seasonal transmission of *Campylobacter* infection occurs prominently and distinctively during winter time in many European countries. Weekly notification rates can increase up to a multiple of the observed notification rates in the weeks before or after the winter peaks. In Switzerland and The Netherlands the notification rates already peaked in the 1st week of January whereas rates for the remaining countries peaked rather in week 2. So far this short-term phenomenon was described in the literature for Germany [3], Switzerland [4, 5] and Luxembourg [6]. For the EU the observation of a winter peak in January was reported for the first time for the years 2012–2014 [1].

Median notification rates over all winter seasons generally increase suddenly in the 1st week of January after a strong decline in the last week of December and do peak in January. The strong decline at the end of December, also observable in countries without a winter peak, could be due to limited access to health care services and reporting delays during public holidays at the end of the year. A study on campylobacteriosis notification data of England and Wales showed that the reporting rate is lower during weeks with a public holiday sometimes resulting in additional reporting in the following week [9]. Annual weekly notification rates of winter peaks showed an increasing trend over the recent years in most affected countries which could be related to the general increase of campylobacteriosis case notifications in Europe since 2005 [1, 2]. The analysis of Austrian, Belgian, German and Norwegian dates of disease onset and of diagnosis revealed that most notified cases show symptoms of campylobacteriosis in the last week of December and the 1st week of January. This observation was recently described for Germany [3]. Hence, winter peaks seen in surveillance data are likely delayed by a few days to 1 week compared to actual peaks of campylobacteriosis in the population when considering the “date used for statistics” of TESSy. These delays are likely caused by time needed for health care seeking, laboratory diagnostics and reporting. When taking into account an average incubation period for campylobacteriosis of 2–5 days, exposure to *Campylobacter* occurs likely around Christmas or New Year for notifications reported in the first 2 weeks of January [3, 5].

### Possible reasons for the seasonal patterns

The sudden increases of weekly notification rates point towards a rapid change in exposure patterns or levels of exposures for campylobacteriosis in winter. Of particular interest appear food- and travel-related exposures around Christmas and New Year. In Finland and Sweden high proportions of travel-related cases (≥50%) are observed in annual surveillance data [1, 2]. Their winter peaks may be partially due to increased travel activities to foreign countries during Christmas and New Year holidays. In Switzerland, travelling abroad during the festive season was associated with almost three-time higher odds for contracting campylobacteriosis [5].

A recent study in Luxembourg identified the consumption of chicken in winter as risk factor for contracting campylobacteriosis and the authors hypothesised that it could be related to the traditional consumption of meat fondue during this time [6]. The consumption of meat fondue or table top grilling during the festive season is popular in Austria, Belgium, Germany, Luxembourg and The Netherlands. In Switzerland, the campylobacteriosis winter peak is associated with the frequent consumption of meat fondue at Christmas and New Year which increased the odds for contracting campylobacteriosis fourfold [5]. At these occasions, possibilities for *Campylobacter* transmission include cross-contamination of cooked meat and/or side dishes by raw poultry meat and individual meat preparation at the table [5, 10]. Hence, individuals are likely to contract campylobacteriosis around Christmas and New Year as a consequence of increased exposure levels to foodborne and travel-related risk factors.

## Limitations

The “date used for statistics” provided by TESSy can vary between reporting countries and could mean the dates of disease onset, of diagnosis, of notification or any other date. The use of a non-standardised reporting date and differences in the national surveillance systems make it difficult to exactly compare the temporal trends of winter peaks among countries. Reporting delays and other surveillance artefacts affecting notification rates of observed winter peaks could not be excluded. Consequently, it should be evaluated whether these peaks represent a true epidemiological trend before investigating possible risk exposures. To our knowledge, there is no scientific evidence on the extent and significance of the consumption of meat fondue or table top grilling for the investigated countries except for Switzerland [5].

## Additional file

**Additional file 1.** Country-specific case numbers and notification rates for winter seasons 2006/2007–2013/2014.

## Abbreviations

ECDC: European Centre for Disease Prevention and Control
EU: European Union
TESSy: The European Surveillance System
