# Supplementary material for: The recurrent campylobacteriosis epidemic over Christmas and New Year in European countries, 2006–2014
Source: BMC Res Notes. 2017 Jul 11;10:266. doi: 10.1186/s13104-017-2587-8 (PMC5504853; doi:10.1186/s13104-017-2587-8)
Supplement: Supplementary file 1 — Additional file 1. Country-specific case numbers and notification rates for winter seasons 2006/2007–2013/2014. [file 13104_2017_2587_MOESM1_ESM.pdf]

Additional file 1

The recurrent campylobacteriosis epidemic over Christmas and New Year in European countries, 2006–2014

BMC Research Notes

Philipp Justus Bless[1,2], Claudia Schmutz[1,2], Daniel Mäusezahl[1,2*]

[1]Swiss Tropical and Public Health Institute, Socinstrasse 57, P. O. Box, 4002 Basel, Switzerland

[2]University of Basel, Petersplatz 1, P. O. Box, 4001 Basel, Switzerland

*corresponding author (daniel.maeusezahl@unibas.ch)

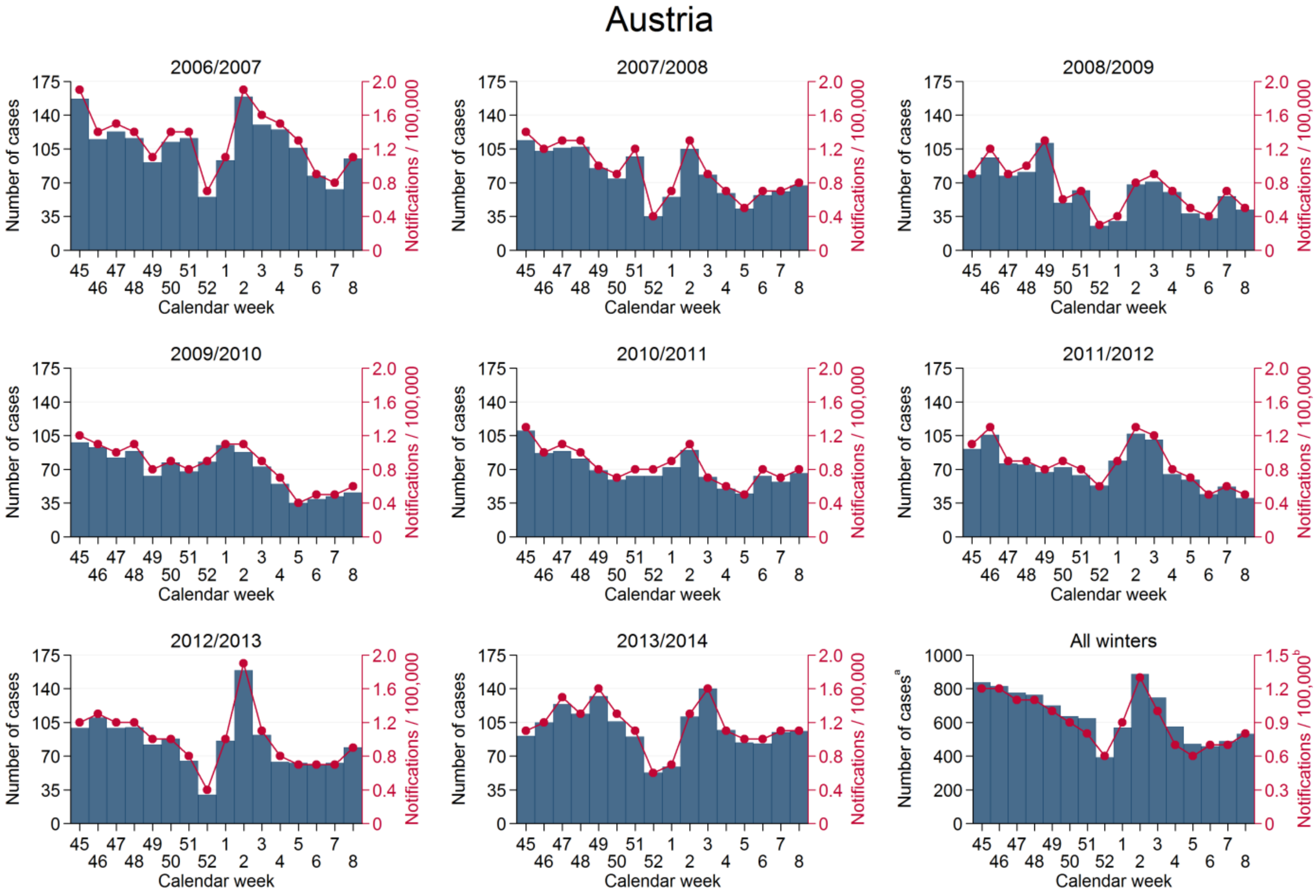

[a]Sum of weekly notifications from winter seasons 2006/2007 to 2013/2014

[b]Weekly notifications per 100,000 population = median of weekly notification rates from winter seasons 2006/2007 to 2013/2014

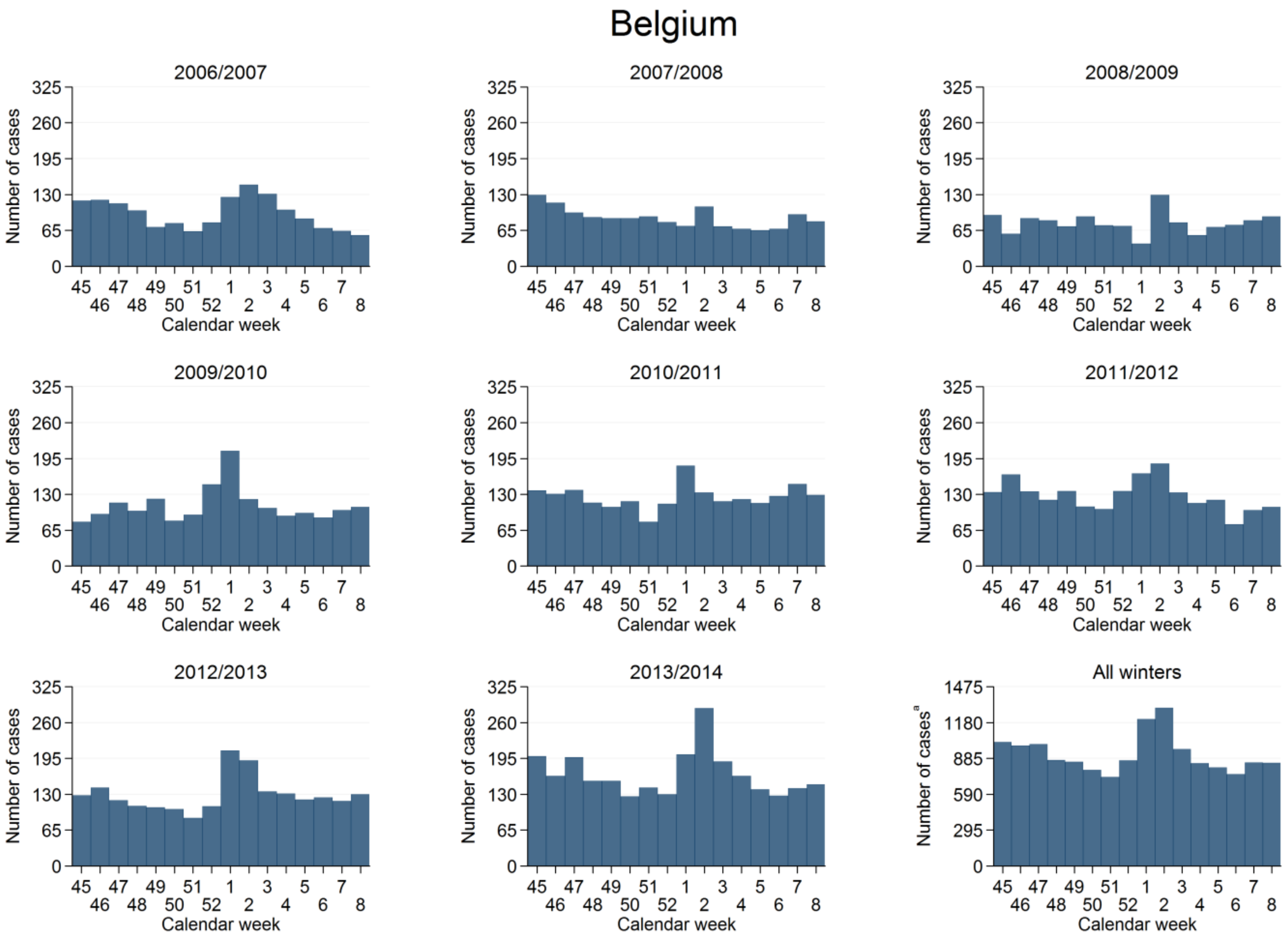

[a]Sum of weekly notifications from winter seasons 2006/2007 to 2013/2014

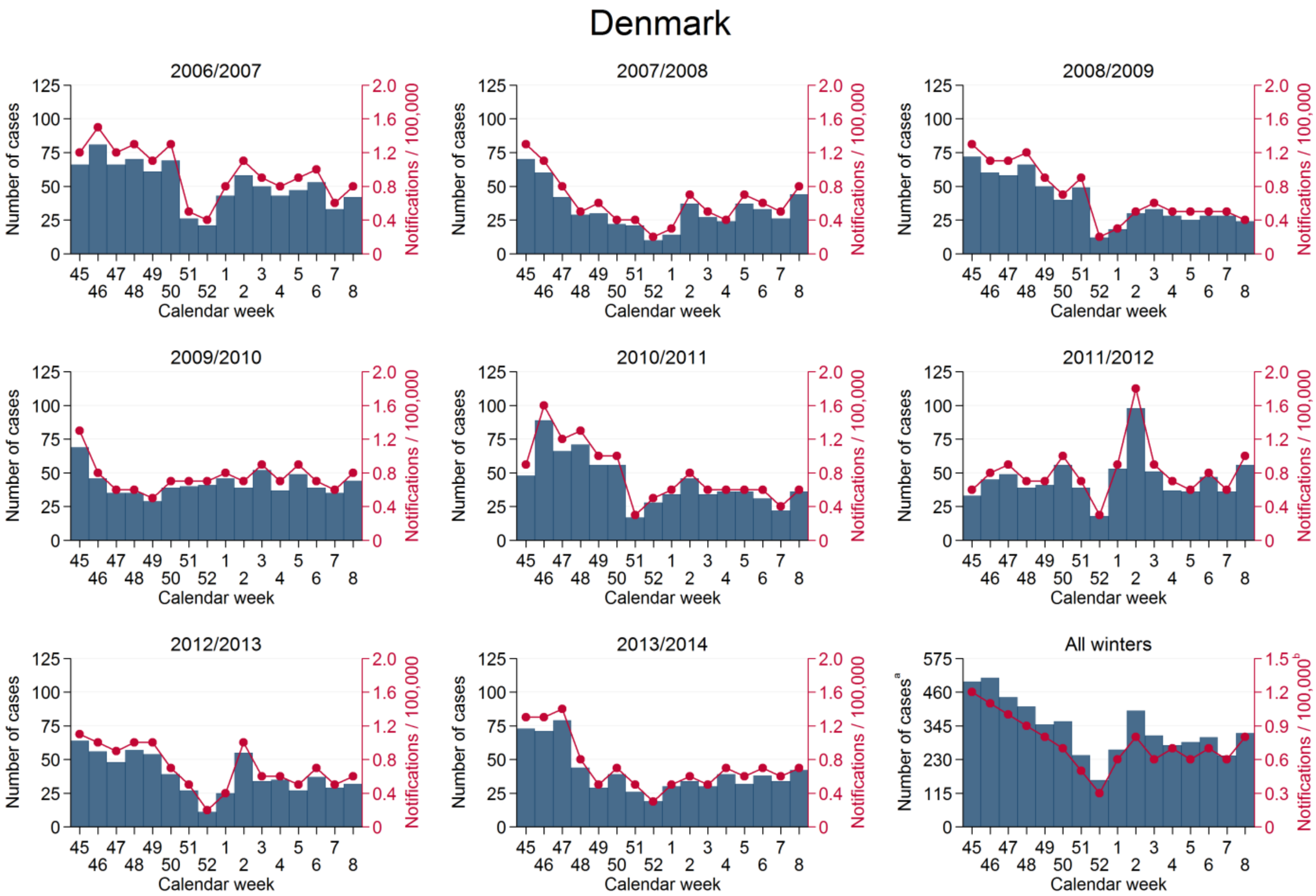

[a]Sum of weekly notifications from winter seasons 2006/2007 to 2013/2014
[b]Weekly notifications per 100,000 population = median of weekly notification rates from winter seasons 2006/2007 to 2013/2014

Finland

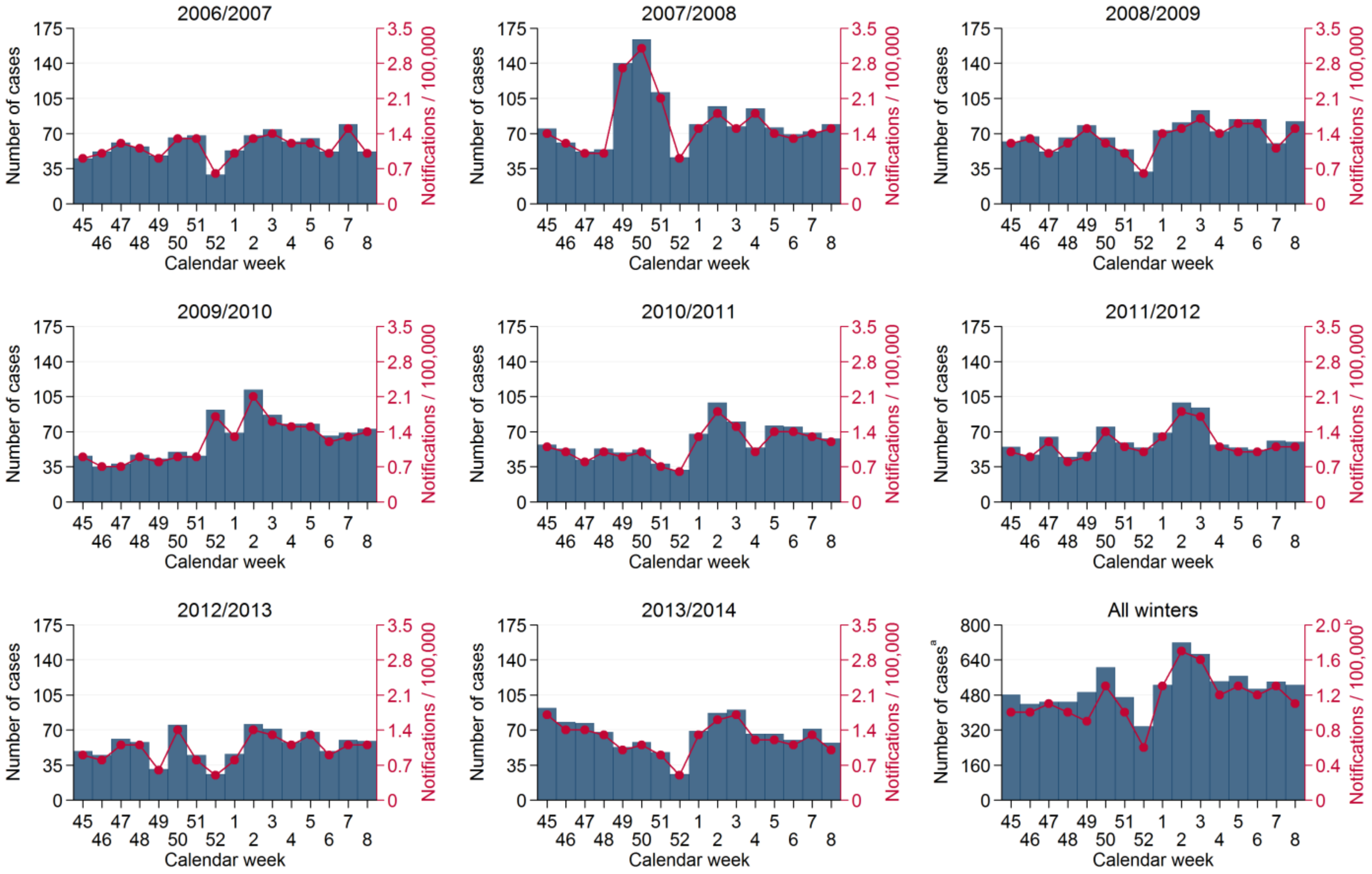

[a]Sum of weekly notifications from winter seasons 2006/2007 to 2013/2014
[b]Weekly notifications per 100,000 population = median of weekly notification rates from winter seasons 2006/2007 to 2013/2014

France

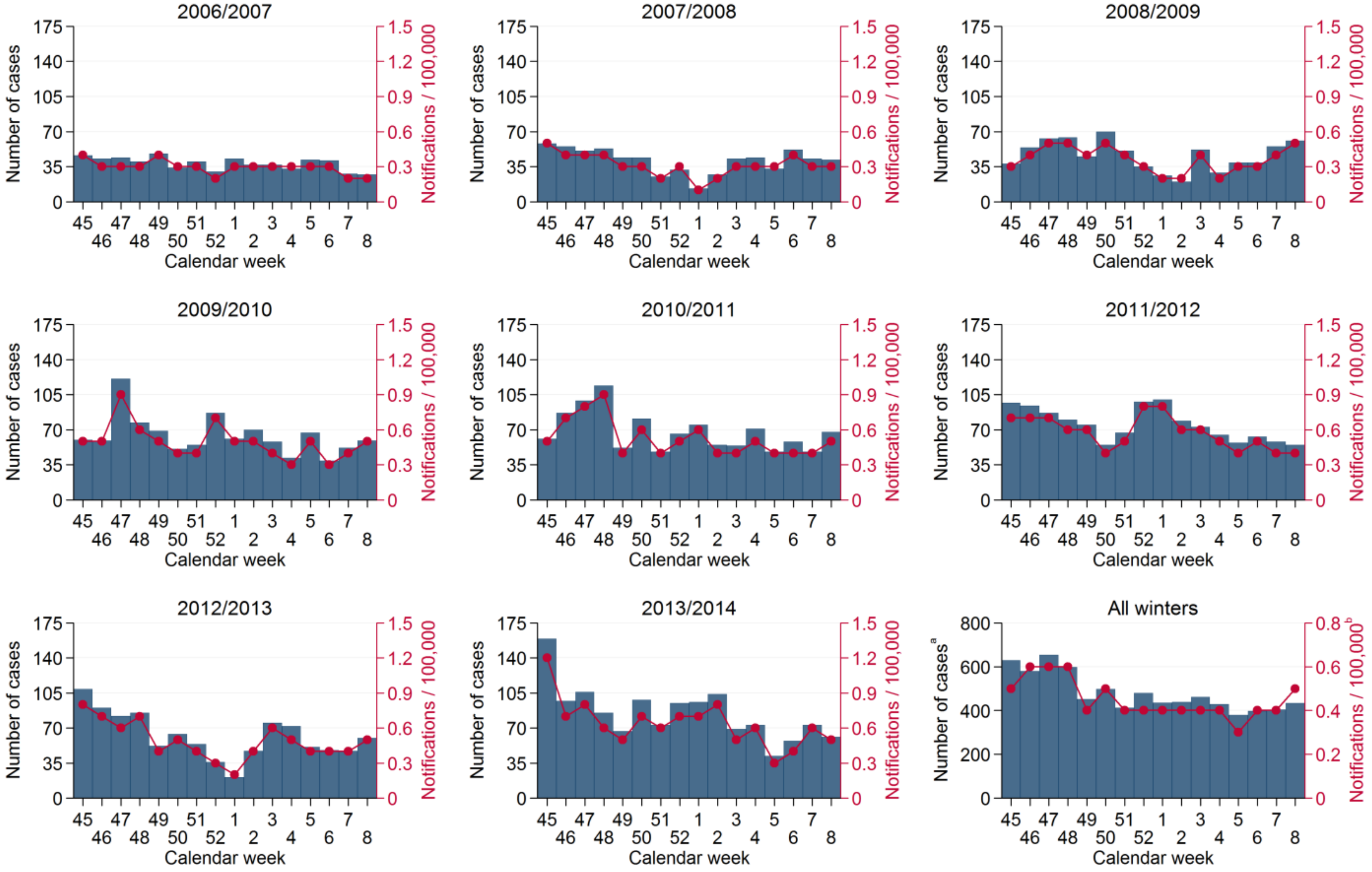

[a]Sum of weekly notifications from winter seasons 2006/2007 to 2013/2014
[b]Weekly notifications per 100,000 population = median of weekly notification rates from winter seasons 2006/2007 to 2013/2014

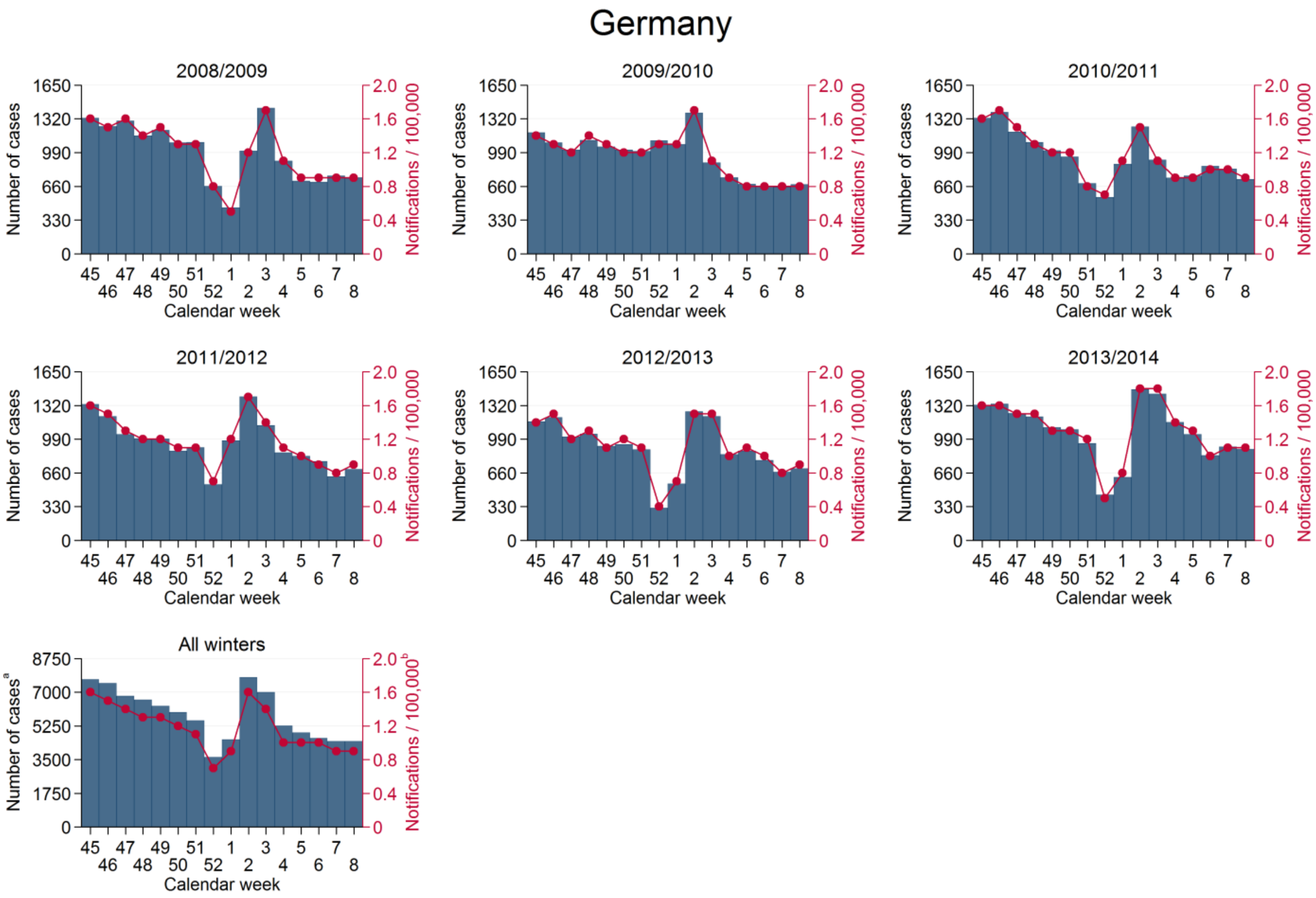

[a]Sum of weekly notifications from winter seasons 2008/2009 to 2013/2014
[b]Weekly notifications per 100,000 population = median of weekly notification rates from winter seasons 2008/2009 to 2013/2014

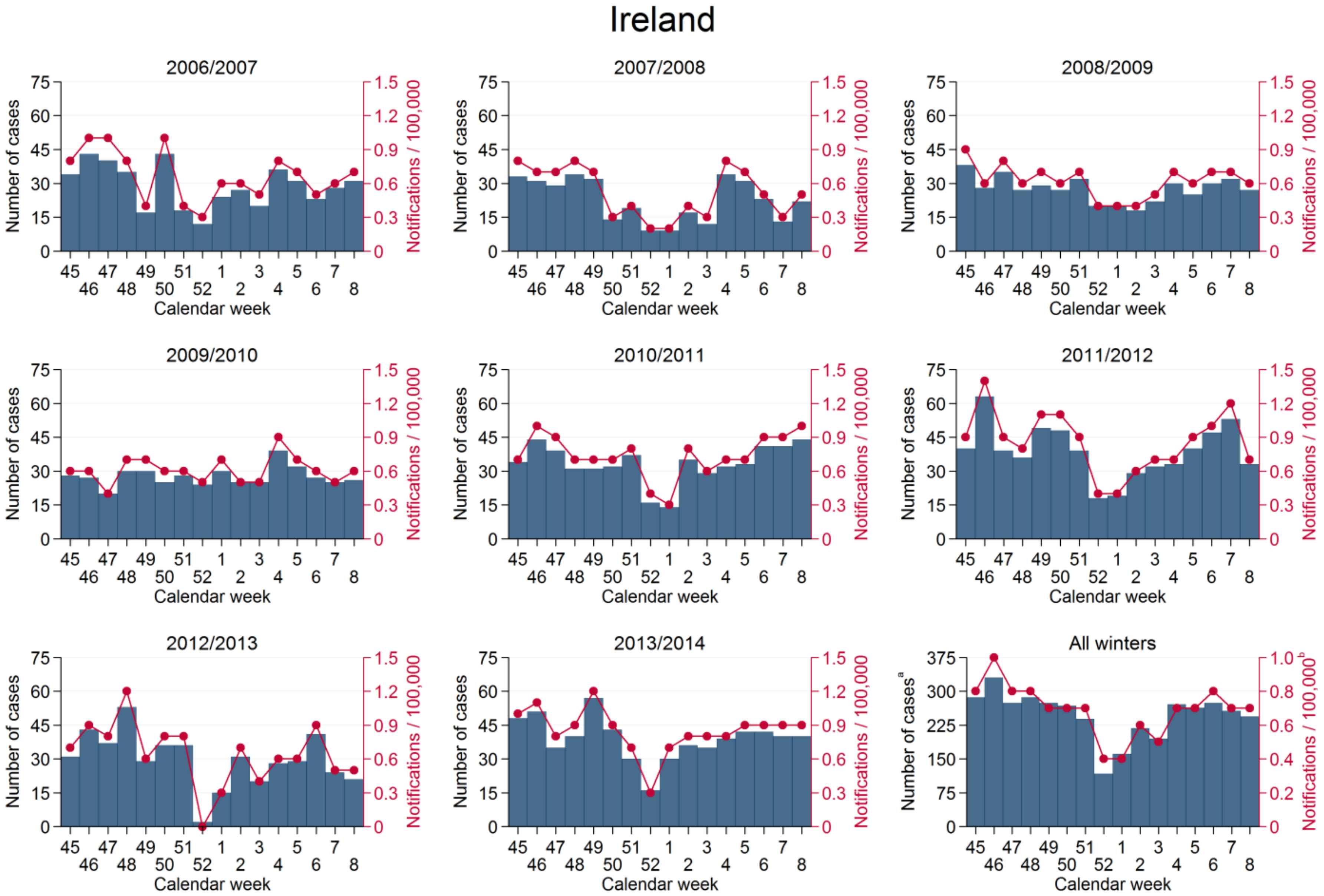

[a]Sum of weekly notifications from winter seasons 2006/2007 to 2013/2014
[b]Weekly notifications per 100,000 population = median of weekly notification rates from winter seasons 2006/2007 to 2013/2014

Italy

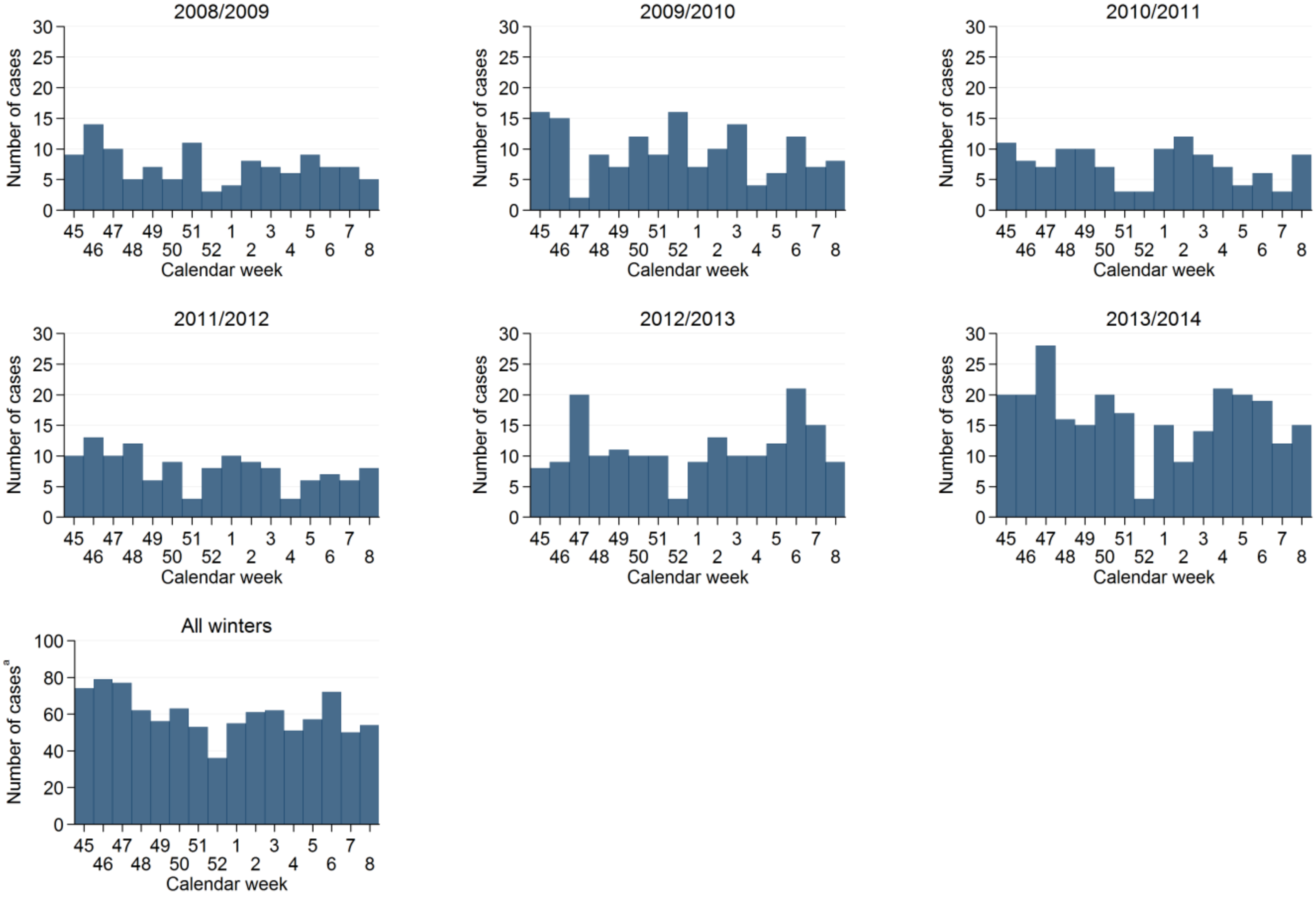

[a]Sum of weekly notifications from winter seasons 2008/2009 to 2013/2014

Luxembourg

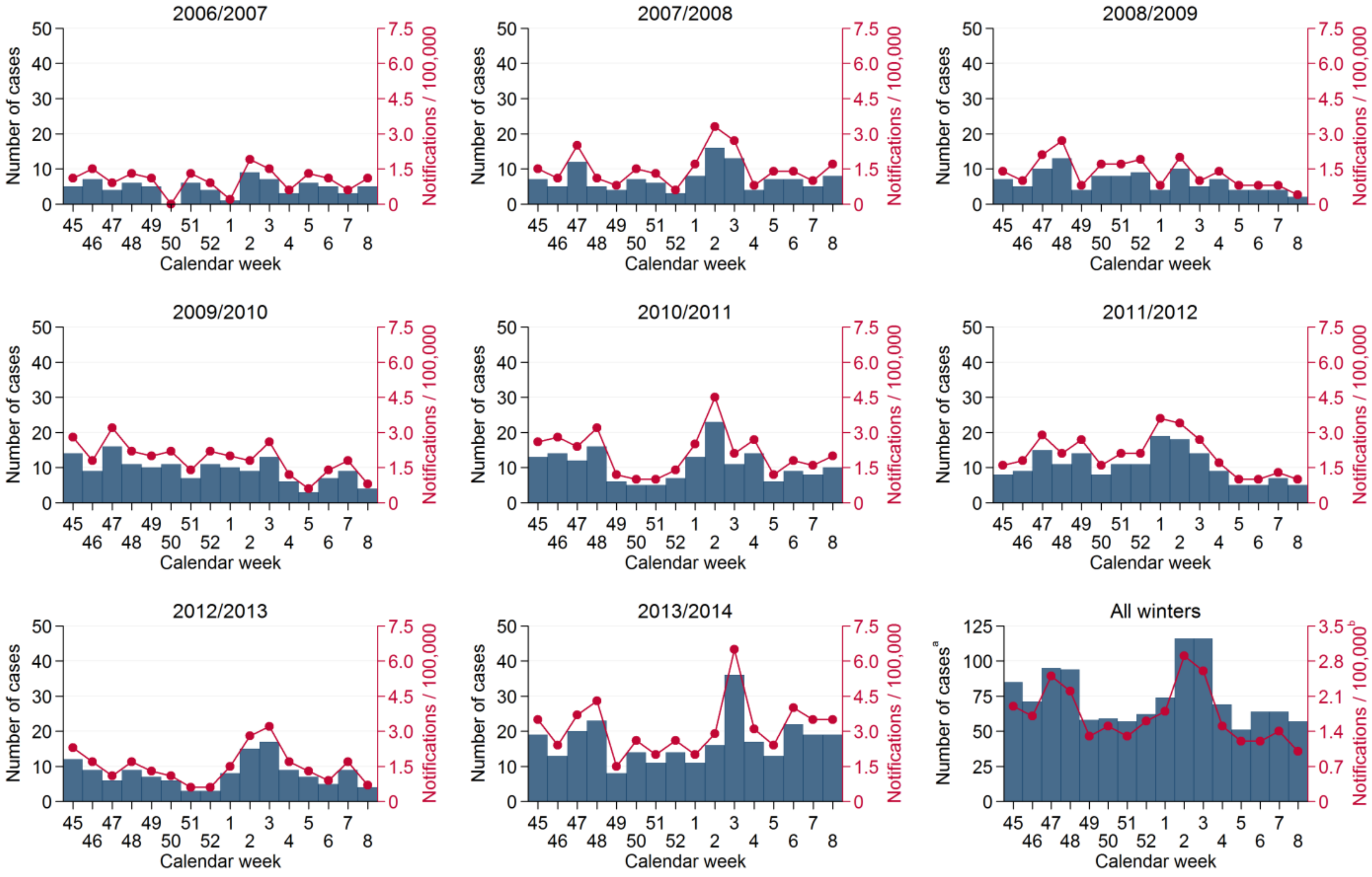

[a]Sum of weekly notifications from winter seasons 2006/2007 to 2013/2014
[b]Weekly notifications per 100,000 population = median of weekly notification rates from winter seasons 2006/2007 to 2013/2014

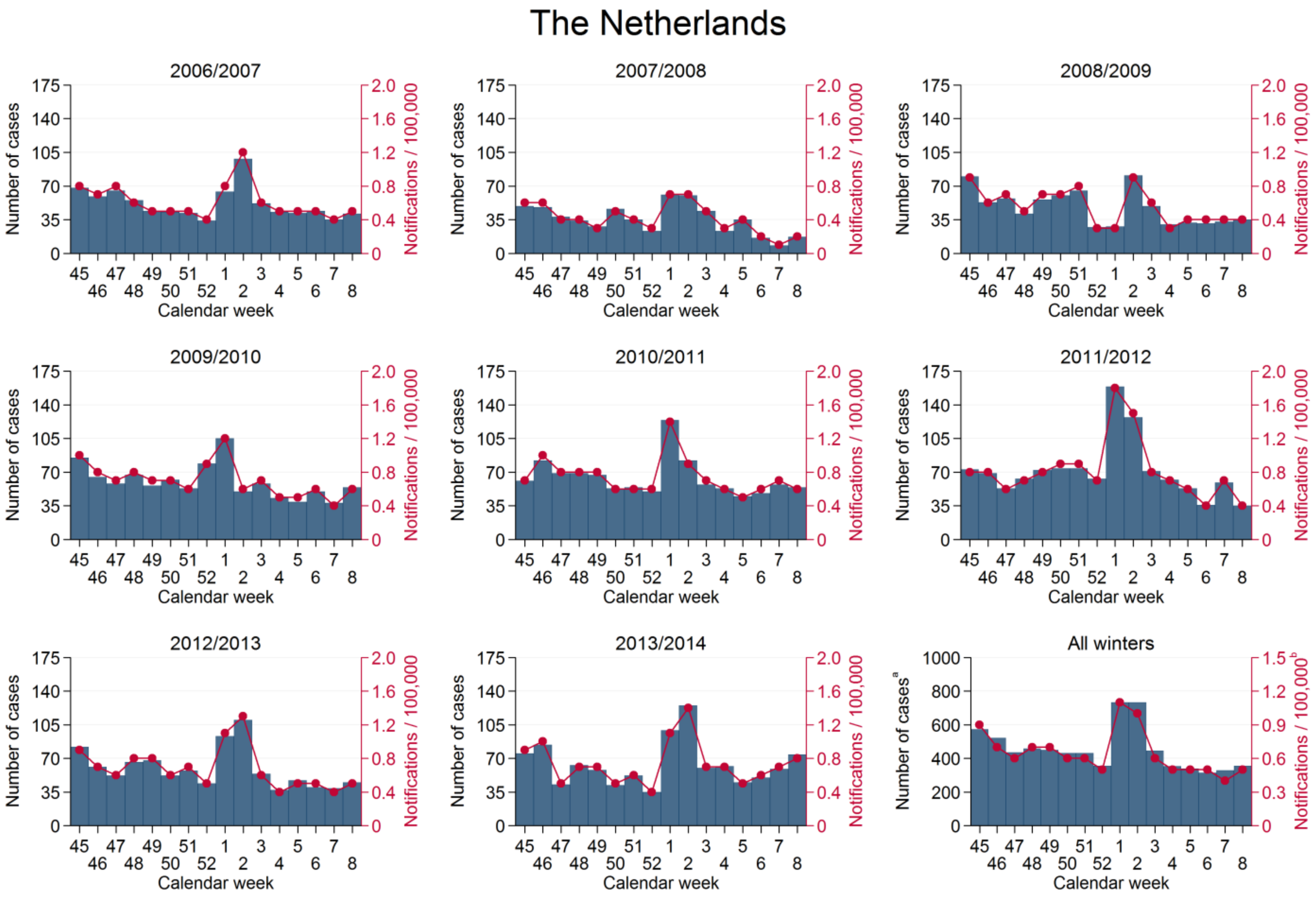

[a]Sum of weekly notifications from winter seasons 2006/2007 to 2013/2014
[b]Weekly notifications per 100,000 population = median of weekly notification rates from winter seasons 2006/2007 to 2013/2014

Norway

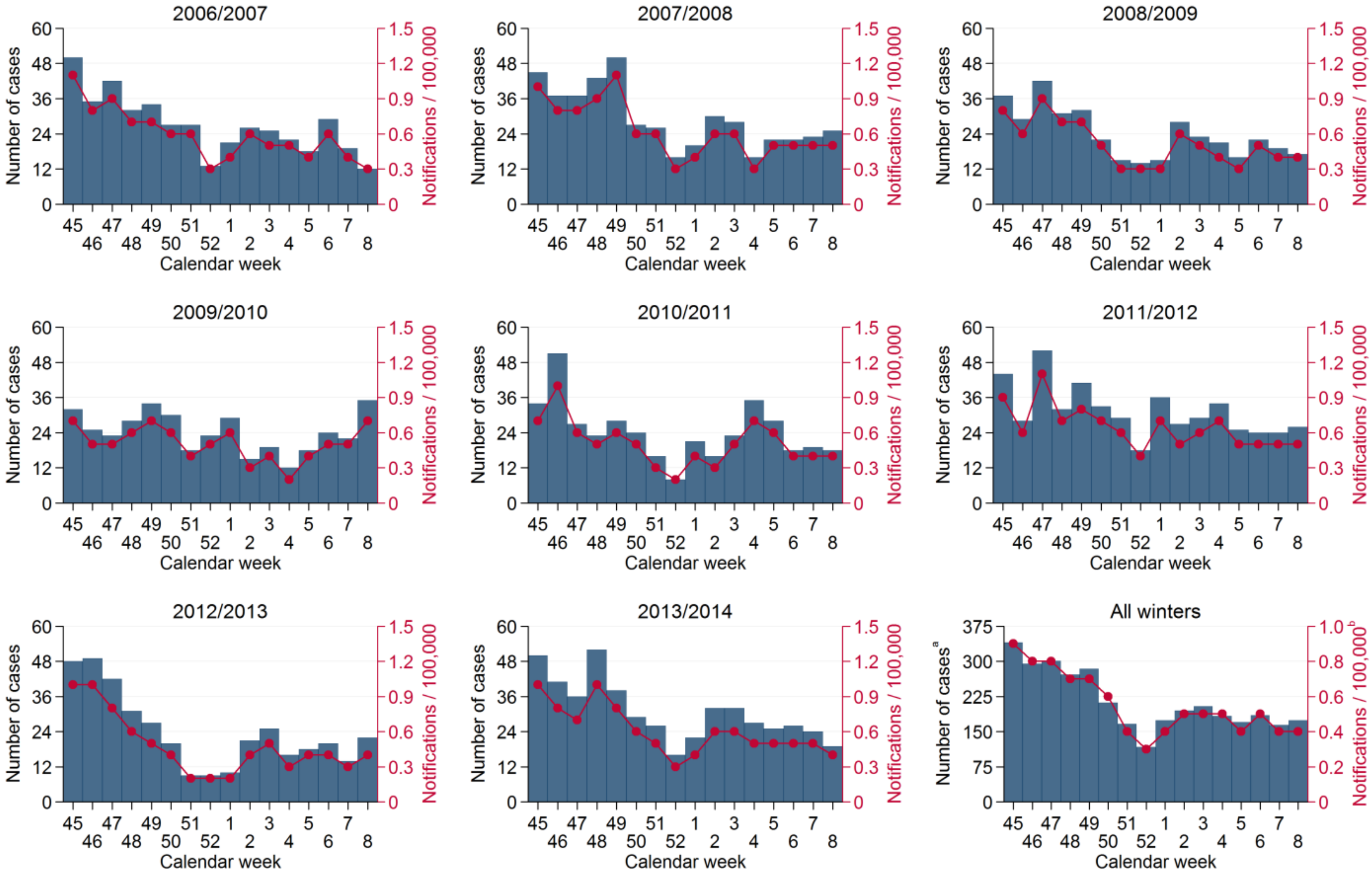

[a]Sum of weekly notifications from winter seasons 2006/2007 to 2013/2014
[b]Weekly notifications per 100,000 population = median of weekly notification rates from winter seasons 2006/2007 to 2013/2014

Sweden

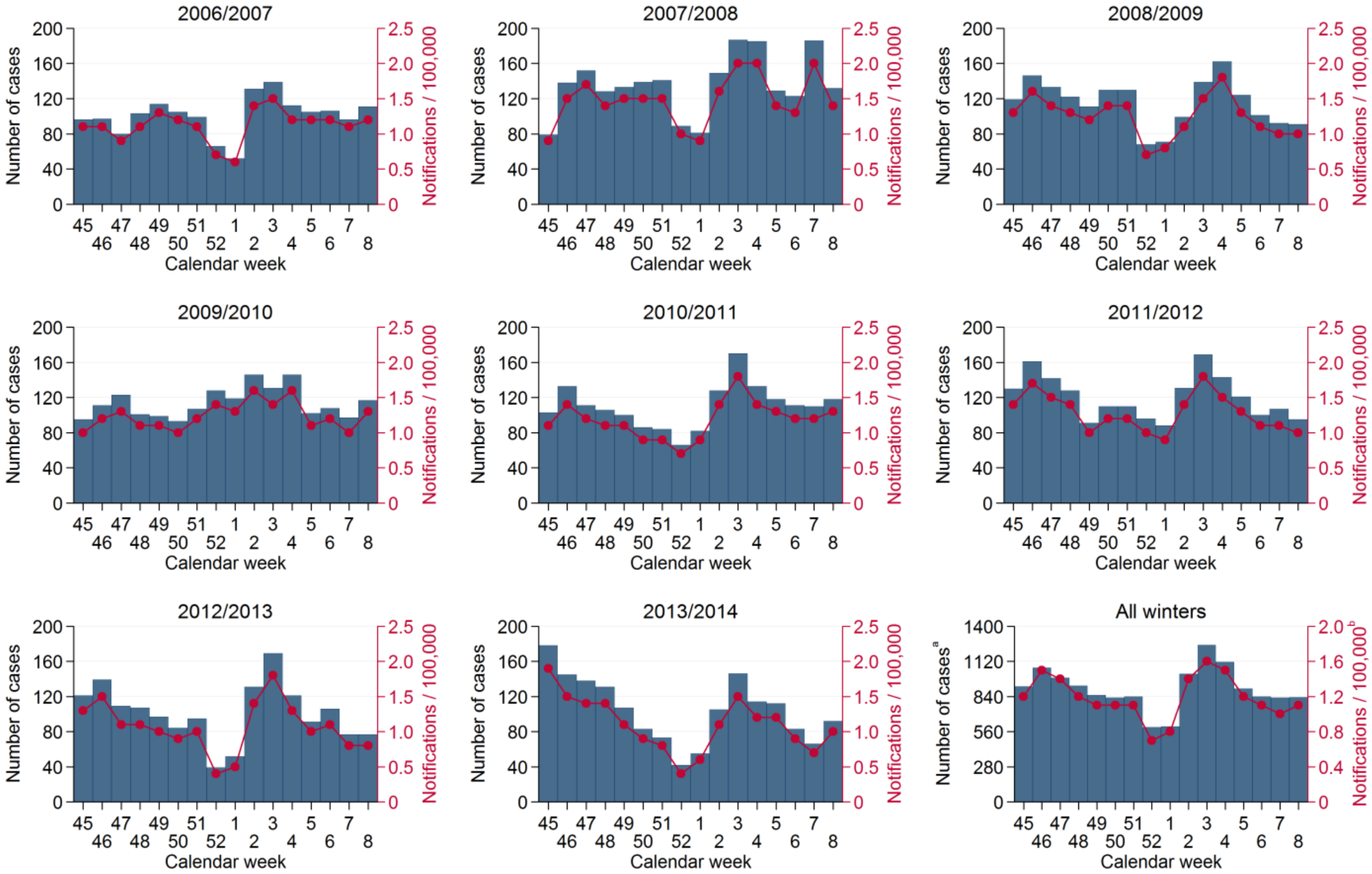

[a]Sum of weekly notifications from winter seasons 2006/2007 to 2013/2014
[b]Weekly notifications per 100,000 population = median of weekly notification rates from winter seasons 2006/2007 to 2013/2014

Switzerland

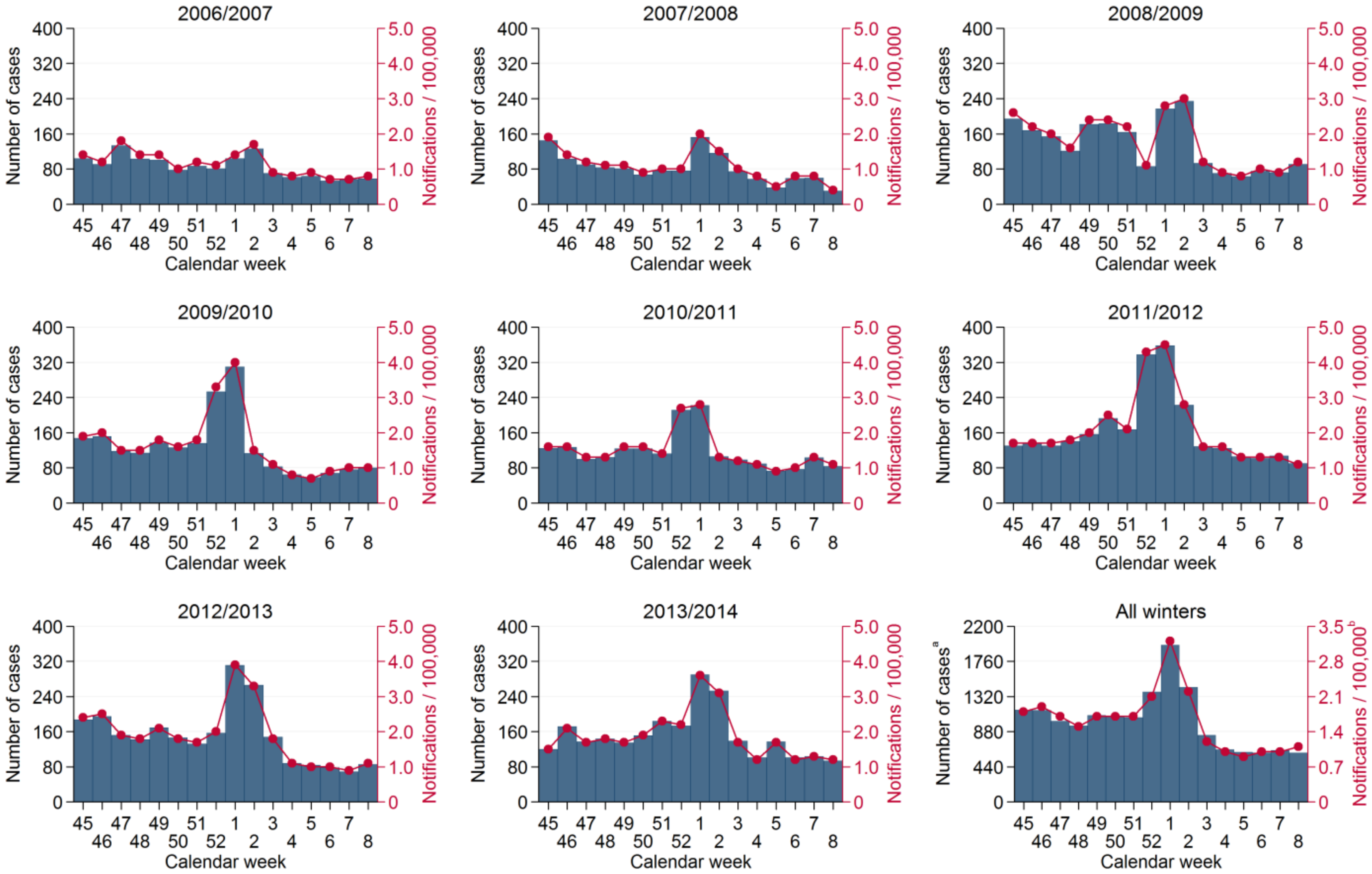

[a]Sum of weekly notifications from winter seasons 2006/2007 to 2013/2014

[b]Weekly notifications per 100,000 population = median of weekly notification rates from winter seasons 2006/2007 to 2013/2014

The United Kingdom

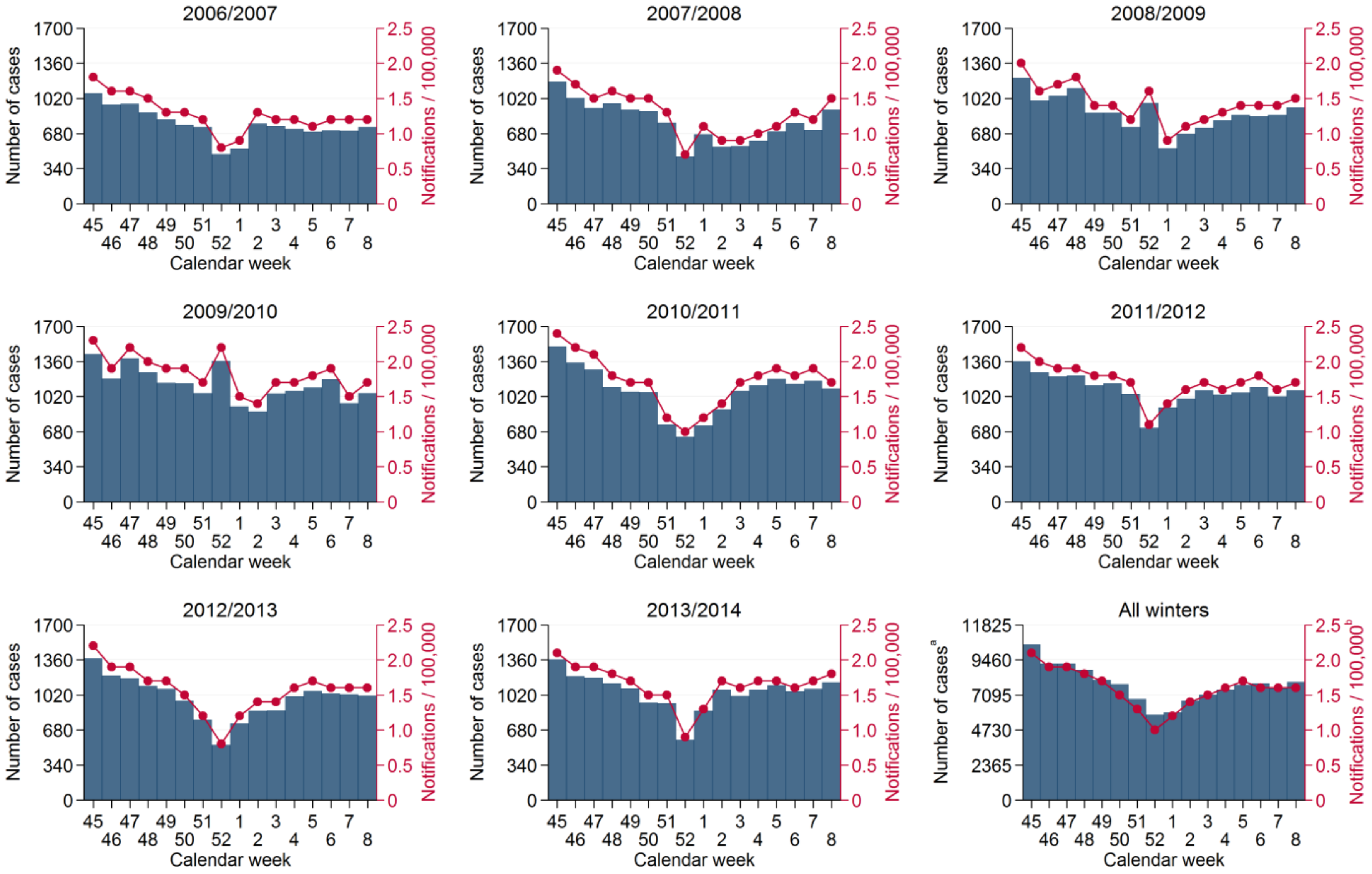

[a]Sum of weekly notifications from winter seasons 2006/2007 to 2013/2014
[b]Weekly notifications per 100,000 population = median of weekly notification rates from winter seasons 2006/2007 to 2013/2014
